# The Amazonian Tropical Bites Research Initiative, a hope for resolving zoonotic neglected tropical diseases in the One Health era

**DOI:** 10.1093/inthealth/ihac048

**Published:** 2022-07-27

**Authors:** Emma Taylor, Elsa Gladys Aguilar-Ancori, Ashley C Banyard, Isis Abel, Clara Mantini-Briggs, Charles L Briggs, Carolina Carrillo, Cesar M Gavidia, Ricardo Castillo-Neyra, Alejandro D Parola, Fredy E Villena, Joaquin M Prada, Brett W Petersen, Nestor Falcon Perez, Cesar Cabezas Sanchez, Moises Sihuincha, Daniel G Streicker, Ciro Maguina Vargas, Ana Maria Navarro Vela, Marco A N Vigilato, Hui Wen Fan, Rodney Willoughby, Daniel L Horton, Sergio E Recuenco

**Affiliations:** U nive rsity of Surrey, School of Veterinary Medicine, Daphne Jackson Road, Guildford, GU2 7AL, UK; Instituto Universitario de Enfermedades Tropicales y Biomedicina de Cusco - Universidad Nacional de San Antonio Abad del Cusco, Cusco, 08003, Peru; Animal and PlantHealth Agency, WoodhamLane, New Haw, Weybridge, Surrey, KT15 3NB, United Kingdom; Laboratório de Epidemiologia e Geoprocessamento, Instituto de MedicinaVeterinária, Universidade Federal do Pará, Castanhal, Pará, 68743-970, Brasil; Berkeley Center for Social Medicine and the Institute for the Study of Societal Issues, University of California, Berkeley, 94720-5670, USA; Berkeley Center for Social Medicine and the Department of Anthropology, University of California, Berkeley, 94720-5670, USA; Instituto de Ciencia y Tecnología Dr. Cesar Milstein, Fundación Pablo Cassará - ConsejoNacional de InvestigacionesCientíficas y Técnicas, Saladillo 2468 (C1440FFX) Ciudad de Buenos Aires, Argentina; Facultad de MedicinaVeterinaria, Universidad Nacional Mayor de San Marcos, Lima, 15021, Perú; Department of Biostatistics, Epidemiology and Informatics, Perelman School of Medicine at University of Pennsylvania, Philadelphia, 19104-6021, USA; One Health Unit, School of Public Health and Administration, Universidad PeruanaCayetano Heredia, Lima, 15102, Peru; Fundación Pablo Cassará. Instituto de Ciencia y Tecnología Dr. Cesar Milstein, Saladillo 2468 (C1440FFX) Ciudad de Buenos Aires, Argentina; Asociaciónpara el Empleo y Bienestar Animal en Investigación y Docencia (ASOPEBAID), Lima, 15072, Peru; U nive rsity of Surrey, School of Veterinary Medicine, Daphne Jackson Road, Guildford, GU2 7AL, UK; Poxvirus and Rabies Branch, National Center for Emerging and Zoonotic Infectious Diseases, Centers for Disease Control and Prevention, Atlanta, 30333, USA; Facultad de MedicinaVeterinaria y Zootecnia, Universidad Peruana Cayetano Heredia, Lima, 15102, Perú; Centro de InvestigacionesTecnologicas, Biomedicas y Medioambientales-CITBM, Universidad Nacional Mayor de San Marcos, Lima, 15081, Peru; Hospital Nacional Arzobispo Loayza, Lima, 15082, Perú; Institute of Biodiversity, Animal Health and Comparative Medicine, University of Glasgow, Glasgow, G12 8QQ, UK; MRC-University of Glasgow Centre for Virus Research, Glasgow, G61 1QH, UK; Instituto de Medicina Tropical Alexander Von Humbolt, Universidad Peruana Cayetano Heredia, Lima, 15102, Perú; Ministerio de Salud, Lima, 15072, Perù; Pan American Center for Foot and Mouth Disease and Veterinary Public Health, Department of Communicable Diseases and Environmental Determinants of Health, Pan American Health Organization, Rio de Janeiro, 25040-004, Brazil; Bioindustrial Center, InstitutoButantan, São Paulo, 05503-900, Brazil; Medical College of Wisconsin, Milwaukee, Wisconsin, 53226, USA; U nive rsity of Surrey, School of Veterinary Medicine, Daphne Jackson Road, Guildford, GU2 7AL, UK; Centro de InvestigacionesTecnologicas, Biomedicas y Medioambientales-CITBM, Universidad Nacional Mayor de San Marcos, Lima, 15081, Peru

**Keywords:** Amazon, bites, indigenous populations, infectious diseases, neglected disease, zoonoses

## Abstract

**Background:**

Neglected tropical diseases (NTDs) disproportionately affect populations living in resource-limited settings. In the Amazon basin, substantial numbers of NTDs are zoonotic, transmitted by vertebrate (dogs, bats, snakes) and invertebrate species (sand flies and triatomine insects). However, no dedicated consortia exist to find commonalities in the risk factors for or mitigations against bite-associated NTDs such as rabies, snake envenoming, Chagas disease and leishmaniasis in the region. The rapid expansion of COVID-19 has further reduced resources for NTDs, exacerbated health inequality and reiterated the need to raise awareness of NTDs related to bites.

**Methods:**

The nine countries that make up the Amazon basin have been considered (Bolivia, Brazil, Colombia, Ecuador, French Guiana, Guyana, Peru, Surinam and Venezuela) in the formation of a new network.

**Results:**

The Amazonian Tropical Bites Research Initiative (ATBRI) has been created, with the aim of creating transdisciplinary solutions to the problem of animal bites leading to disease in Amazonian communities. The ATBRI seeks to unify the currently disjointed approach to the control of bite-related neglected zoonoses across Latin America.

**Conclusions:**

The coordination of different sectors and inclusion of all stakeholders will advance this field and generate evidence for policy-making, promoting governance and linkage across a One Health arena.

## Introduction

The Amazon Basin is home to an estimated 38 million people with rural communities in these rainforest landscapes sharing similar cultural, socioeconomic, topographical and environmental issues. The Amazon Basin is one of 10 global hotspots for neglected tropical diseases (NTDs).^1,2^ Nine countries are included in the Amazon basin: Bolivia, Brazil, Colombia, Ecuador, French Guiana, Guyana, Peru, Surinam and Venezuela. The list of NTDs is diverse, and includes parasitic, bacterial, viral, fungal infections and snakebites that disproportionally affect the poorest of communities across the world, leading to physical and cognitive developmental disorders and, in some cases, death.^3^ Existing evidence focuses on drivers associated with each disease, but vectors are found across regions, and individual diseases debilitate populations synergistically. The true burden of NTDs across the Amazon Basin region varies significantly, with obvious data gaps identified in establishing the proportion of communities in the region affected by NTDs, which will inevitably impact the allocation of resources and the success of control and eradication programmes. Indigenous communities are vulnerable to the introduction of communicable diseases and continuously experience the burden of NTDs with access to health facilities being impeded based on ethnicity, wealth and education status. With support of the Pan American Health Organisation (PAHO), the Health of the Indigenous People of the Americas Program was created, with the aim of improving the health of indigenous people, by working in collaboration with the communities themselves.^4,5^ However, there is still a need for transdisciplinary solutions to the problem of animal bites including mammals and insects, and vector-borne disease leading to disease in Amazonian communities.

Here, we discuss the launch of The Amazonian Tropical Bites Research Initiative (ATBRI), a new initiative aiming to fill the gaps left by the current programmes and work with them to develop more comprehensive and inclusive solutions for the Amazonian countries. The research and activities of this new network will create awareness within the communities, public health and veterinary institutions of the burden of NTDs, and the efforts being made to control them. We consider four conditions concentrated in the Amazon basin (i.e. rabies, snakebite envenomation, Chagas disease and leishmaniasis) mediated mainly via animal bites but also pathogen-contaminated faeces.

### Rabies

Dog-mediated rabies in humans has been significantly reduced in the Americas, by an estimated 99% in Latin America; however, reports of cases in dogs are still being received and so the threat from terrestrial carnivore populations remains.^6–8^ Even although the full burden of dog bites and dog-mediated human rabies has not been quantified in Latin America, some communities in these Amazonian countries report the highest dog bite rates in the world^9^ and some areas are experiencing a sharp increase in dog rabies cases after COVID-19–associated disruptions of mass dog vaccination campaigns and surveillance activities.^6^

Rabies from haematophagous bats (principally the common vampire bat, *Desmodusrotundus*) causes significant economic losses through infection of cattle and other agricultural species of value. Rabies outbreaks in the Amazon also cause devastating losses of human lives, sometimes killing tens of individuals in small communities. The true frequency of these larger mortality events remains poorly understood and single mortality events are probably even more difficult to detect.^10^ Human risk of vampire bat bites may be rising due to the increasing residency of humans in areas with limited alternative prey for bats (as observed in fishing communities in riversides of Brazil) or as a consequence of human-driven depletion of alternative wildlife prey by mining, deforestation or hunting.^11,12^

Primary prevention of rabies involves pre-exposure prophylaxis of human and animals, and for humans, avoiding animal bites through health education. Health education programmes should also teach about the importance of immediate wound washing with soap and water in the event of a bite. Secondary prevention involves the use of rabies biologics for post-exposure prophylaxis (PEP) of humans who have experienced bites, and guidelines for cleansing bites with soap and water. Prevention efforts in Europe and North America, which focus on wildlife rabies, proved the value of oral rabies vaccines, such as RABORAL V-RG, as tools for controlling outbreaks in wildlife carnivore populations including foxes in Europe and racoons in North America.^13,14^ By contrast, in most Amazonian regions, vampire bats are the most frequent source of human rabies, raising a distinct challenge for scalable vaccine delivery.^15^ In the absence of bait systems, efforts are under way to develop virally vectored recombinant rabies vaccines with variable degrees of capacity for self-dissemination among bats as well as systems to aerosolise vaccines within bat roosts.^16,17^ While mathematical models indicate such approaches could outperform current approaches to rabies control by culling vampire bats, deeper understanding of the safety and potential ecological consequences of such vaccines will be required before large-scale deployments are possible.^18,19^

The Meeting of Rabies Program Directors in the Americas (REDIPRA), organised by the PAHO, has been held approximately every 2 y since 1983.^20^ These REDIPRA meetings are attended by directors of national rabies control programmes from the Ministry of Health (MoH) and the Ministry of Agriculture (MoA) from representative countries within the Americas. The aim of these meetings is to review the current strategies for the prevention of human rabies, and to produce further recommendations towards eliminating dog-mediated human rabies, and reduction of rabies transmitted via wildlife species. In 2018, a total of 33 549 cases of wild and domestic animal bite notifications were reported to the Brazilian Information System on Notifiable Diseases; Sistema de Informação de Agravos de Notificação (SINAN) database.^21^ Established in 1969 by PAHO, the Sistema de Information Regional para la VigilanciaEpidemiologica de la Rabia (SIRVERA) is a database for both human and animal rabies cases. In 2021, seven cases of dog-mediated human rabies were reported to SIRVERA.^22^ The availability of these data is imperative for establishing the epidemiological situation for rabies, and is fundamental to informing control strategies.

### Snakebite envenomation

The Latin American Network of Public Antivenom Manufacturing Laboratories (RELAPA, Red Latinoamericana de Laboratorios Públicos Productores de Antivenenos), in collaboration with the WHO, recommended to include snakebite envenoming as an NTD and categorise it into Category A, diseases listed as receiving full resources from the WHO.^23^ A global strategy to reduce mortality and disability from snakebite envenoming by 50% before 2030 has been developed.^23^ Additionally, the 71st World Health Assembly (2018) proposed to resolve the global issue of snakebite envenoming through immediate action.^24^ Snakebite envenoming is estimated to cause up to 138 000 deaths worldwide per year, with up to 70% of those bitten choosing to seek traditional medicine as treatment, with 400 000 experiencing permanent disability.^25^ Venom toxins of Viperidae (viperids), a family of venomous snake species, for example, can cause local tissue damage and systemic effects, such as coagulation disturbances, bleeding and acute kidney injury, while some may induce neurological, automatic and myotoxic diseases.^26^ Differential diagnosis is also required for exposure related to bites from *Bothrops* snake species, commonly known as fer-de-lance (French), pit viper (English) and Terciopelo Cuatro Narices (Spanish). While antivenom exists for *Bothrops* bite cases, there are few studies assessing serum efficacy and potency, safety and sustainability affecting the overall quality of the treatment.^23,27^ It is recognised that global access to antivenom is low for both quantity and quality, with an estimated <5–20% of patients treated. Within Amazonian countries, some public laboratories that manufacture antivenom need support for research and development to upgrade their processes and quality control methods to international benchmarks. Where funded, scientific advances in the field have included a deeper knowledge of snake venoms, including the biochemistry, proteomics, immunology and bioinformatics.^28^ This knowledge should be directed to assess the efficacy and safety of new combinatorial antivenom product.^29^ A network of public laboratories that manufacture antivenoms in Latin America was established in 2019 (RELAPA) to increase the availability and access to effective and safe antivenoms throughout Latin America.^24,26,30^

### Vector-borne NTDs

Chagas disease is a zoonotic disease caused by the parasite *Trypanosoma cruzi*, with an estimated 8–11 million people infected in the region, and a further 25 million at risk of developing the disease.^31^ The parasite can be transmitted directly from blood-sucking triatomine insects commonly known as ‘kissing bugs’ via stercorarian transmission: the infected bug defecates while feeding depositing infective parasites over the skin that use the bite hole or any mucosal membrane to enter the bloodstream. Other transmission routes are consumption of fruits contaminated with a parasite-carrying insect faeces, organ transplants, blood transfusion and congenitally from mother to foetus across the placenta.^32^ Even although the parasite is not transmitted directly through bites, it is the biting, and the search for bloodmeals, that put humans and triatomine insects in contact and increases the risk of Chagas disease. In 1991, the Southern Cone Initiative to control and eliminate chagas disease was launched, with the cooperation and support of PAHO, with governments from two Amazonian countries taking part (Bolivia and Brazil). Under the initiative, 2.5 million homes have been treated with pyrethroid, a long-lasting insecticide, and poor housing in areas of poverty has been improved to help eliminate environments that support triatomine insects.^33,34^ In South America, through the Southern Cone Initiative,^35^ national Chagas disease programmes, mostly based on indoor residual spraying, were launched to eliminate vectorial transmission. Nowadays, multiple countries in the Amazon basin have successfully controlled or eliminated *T. cruzi* transmission from large urban areas.^35^ However, transmission is increasing in new human settlements in the Amazon, and the constant movement of human populations poses a continuous risk for the expansion of areas with active *T. cruzi* transmission.^35^

Leishmaniasis is a group of diseases that are caused by protozoa transmitted by the bite of infected female sand flies. Over 12 million people in more than 98 countries worldwide are affected by leishmaniasis, with >1.5 million new cases of cutaneous leishmaniasis (CL) each year.^36^ It is challenging to estimate the burden of leishmaniasis in the Amazon Basin; however, estimates taken for the 2004–2008 Global Burden study suggest a total of 56 936 cases for the Amazon region.^37^ The Amazonian region of Madre de Dios, which connects Peru, Brazil and Bolivia, has seen a change in land use due to the increase of agriculture and logging activities. The combined effects of environmental and land use changes, along with environmental disruption caused by climate change, increase the risk of transmission of parasitic diseases to humans, such as leishmaniasis.^38^ It has been documented that the majority of human infections are acquired during the clearing of large areas of forest, disrupting the natural land use and human occupation of new areas, changing the behaviour of the vector species.^38^

### Barriers to disease control

A One Health approach, which emphasises an interdisciplinary, cooperative approach to veterinary and human health, is recognised as an effective way of managing emerging infectious disease threats. There are, however, some fundamental challenges associated with the success of a One Health approach, including socioeconomic and political barriers to collaboration.^39^

The emergence of new pathogens has been attributed to change in habitat biodiversity, deforestation and an increase in wildlife–human interaction.^40^ Certainly, fluctuations in human population density may result in an increase in the exposure risk of susceptible human and domestic animal populations to pathogens circulating in human, domestic animal and wild animal populations.^41,42^ This can be observed in communities that migrate from The Andes to the forests.^43^ For example, the presence of gold mining sites and their associated deforestation support the spread of NTDs including rabies, due to bites from the common vampire bat.

Political instability can result in gaps in governance, redirection of funding specific to NTD control and eradication, to other diseases, disruption of infrastructure and loss of healthcare access for local communities. Socioeconomic instabilities are also likely to facilitate the spread of infectious diseases. The rise in illegal mining, and disruption of existing disease control and surveillance activities, has perpetuated the humanitarian crisis in Venezuela. Specifically, this has been noted to have a disproportionate effect on vector-borne diseases, with a significant increase in the number of cases of Chagas disease and leishmaniasis. The humanitarian crisis experienced in Venezuela has resulted in populations migrating to Brazil, Ecuador and Colombia, increasing the risk of spread of vector-borne NTDs.^44,45^

Additional threats may emerge beyond those caused from direct exposure. An example of this is vampire bat saliva and faeces, which may contaminate bite wounds and contain a considerable diversity of potentially zoonotic viruses, bacteria and protozoans, including herpeviruses, reoviruses, deltaviruses, astroviruses, coronaviruses, picornaviruses, *Bartonella* spp., *Mycoplasma* spp. and *T. cruzi*, the agent of Chagas disease.^46–48^ While novel pandemic and emerging infectious threats receive considerable attention and funding (e.g. Ebola, Zika, influenza and the new coronavirus outbreak), endemic NTDs remain overlooked. It is suggested in the 2019 G-Finder Report that funding available for NTD research is decreasing.^49^ Furthermore, the need to establish a new funding model, capturing the individual needs of NTDs, particularly the redirection of funding toward research and development, is recognised.^50^

### Mental health and bite-related NTDs

For those individuals that receive PEP, exposure to some NTDs, along with malnutrition and cognitive limitation such as parasitic anaemia in adolescent girls, can have lasting implications on the quality of life.^51^ Disease exposure may lead to stigmatisation, trauma associated with the event and limitations to work, the latter resulting in a relentless cycle of disease and poverty.^52^ Studies have assessed long-term psychological disorders and have identified post-traumatic stress disorder (PTSD), hysteria, delusional disorders, acute stress disorders and depressive disorder as being associated with snakebite.^53,54^ Individuals who have experienced snakebite display more symptoms associated with PTSD, and depressive disorder, than individuals who have not been bitten.^53^ Similarly, those have experienced dog bites report PTSD, leading to cognitive and behavioural changes,^55^ and individuals will often go on to exhibit fear around unknown dogs. There are very limited data on the psychological impact of bat bites, which may be because those who experience bites become accustomed to being bitten by bats. This was reflected in a study in Peru, where despite 90% of the community being bitten by a vampire bat, individuals displayed a high level of misinformation regarding the risk of rabies exposure.^56,57^

Additionally, there is a recognised mental health burden on individuals who do not receive treatment after exposure, as seen in individuals experiencing leishmaniasis, who report an increased risk of mental illness and reduced quality of life.^58^ Further, those individuals with Chagas disease have to learn to cope with the emotional distress associated with living with the condition. However, while there are just two available treatments (benznidazole and nifurtimox) available for treatment of Chagas disease, the administration of these medications allows for the treatment of the acute phase, and chronic phase in children, and women of child-bearing age. Additionally, published guidelines for the diagnosis and treatment of Chagas disease recommend the prescription of trypanocidal treatments for adults with acute infection. Lastly, it is important to note that treatment is free in countries endemic for the disease.^59,60^

Now more than ever, there is an urgent need to renew momentum towards achieving targets set under the NTD 2030 roadmap. Progress made over the last decade has identified that a multisectoral approach is required to accelerate control and elimination of all 17 NTDs. Critical actions to support reaching the 2030 targets have been identified for each NTD. Specifically, targeted for elimination as a public health problem (Chagas disease, visceral leishmaniasis, rabies), targeted for control (CL, snakebite envenoming), each disease requires its own individual combination of actions to meet the associated disease targets. However, all NTDs are included in the four cross-cutting themes; the integration of interventions that are common across several of the diseases, mainstreaming healthcare systems to improve the quality of NTD management to support universal health coverage, coordination with relevant programmes, improving and strengthening national healthcare systems capacity to deliver interventions and increasing regional and global support for programmes.^61^

## The proposed network

The ATBRI met in Lima in 2019 to bring together a focused group of participants from public health agencies and academic institutions. The attendees were from Centro de Investigaciones Tecnologicas, Biomedicas y Medioambientales (CITBM, Peru) and PAHO. More institutions from Amazonian countries were invited but were not able to attend. Sixty-seven participants with expertise in the management and impact of zoonotic and non-zoonotic disease on public and veterinary health from countries across Latin America, the USA and the UK participated in the first meeting. The meeting was held by CITBM and Universidad Nacional Mayor de San Marcos, with support from the Peruvian National Council of Science and Technology (CONCYTEC).

### Aims of ATBRI: mission statement

The aim of the network is creating transdisciplinary solutions to the problem of animal bites (mammals, snakes, insects and vector-borne) leading to disease in Amazonian communities, through research, technological innovation and awareness. The network will prioritise an extremely neglected topic and thus support the development and investment of an early response system for zoonotic diseases that enables early detection of outbreaks of novel and re-emerging threats.^62^ Investing in fortification of early detection surveillance systems for NTDs can help us to prevent pandemic spread of novel zoonoses, which high- to low-income countries would benefit from.

### ATBRI structure

Participants agreed on annual general meetings, with additional meetings of subgroups throughout the year. The general meeting can be held in association with official bite-related meetings from WHO/PAHO such as REDIPRA with established One Health agendas. The need to define roles and responsibilities was also identified, as well as proposing the placement of a Project Manager to oversee the network. Initial site visits to collaborating centres should also take place in the first years following the initial network meeting. The ATBRI group also identified other networks with One Health agendas where members could represent the network. Core priorities of ATBRI were identified via a list of focused questions and actions, with proposed methods and detailed gaps in knowledge they would address (Tables 1 and 2).

**Table 1.** Priorities identified by members attending the first ATBRI meeting, and methods and justification proposed to address them

Question	Method	Justification
What does the community perceive as being the priority health concern, and how does this correlate to country priority?	Use of pilot information, via construction of a literature review.	To establish needs and where the same issue exists in different regions.
	The identification of laboratory access and documentation of network members’ expertise areas.	Support the linking of laboratories to create subgroups. Will help to identify what the capacity is in different countries during outbreaks.
	Community participation and consultation. Direct collaboration of social scientists in field research and research design.	Community-led network to support the inclusion of those communities who are currently neglected. Timely access of exposed population.
What are the pathogens present per region?	Laboratory diagnostics, characterisation, assays.	Sample acquisition and sharing, which is currently a challenge in Latin America.
How to control the venomous animal and vector population with community involvement and buy-in that is sustainable?	Animal and vector identification, community participation and behavioural change and perception of threats of bites and zoonotic disease.	Support towards NTD roadmap 2030, which focuses on the importance of a sustainable approach to disease control.
How to integrate public health sector (MoH) and animal governmental agencies (MoA) to encourage a One Health approach?	Ensure routine communications via active working groups.	Continuation of a transparent and collaborative approach.
	Establish data-sharing agreements.	

**Table 2.** A defined list of activities to be achieved to develop the progression of identified priorities

	
*Communication*	1. Identify target audience that the network needs to incorporate.
	2. Hold an annual meeting anywhere in the Amazonian countries.
	3. Organise a committee that is made up of multiple disciplines, avoiding a hierarchical approach.
	4. The development and maintenance of a website.
	5. Seek to produce publication of the results of this initial workshop in two peer reviewed journals (e.g. *Public Health*).
*Knowledge Exchange*	1. Define capabilities in the different countries regarding bite-related diseases.
	2. Recruit additional collaborators.
	3. Identification of other institutes and key contact points.
	4. Determine training needs in veterinary, public health and anthropological sciences.
*Environment*	1. Identify population dynamics of host for specific disease.
	2. Identify prevalence and incidence of pathogen in environment.
	3. Identify prevalence and incidence of immunity in local community.
	4. Identify spatio-temporal dynamics that support the circulation of pathogens.
	5. Identification of risk factors for diseases.
*Health, Education, Social Sciences and Community engagement*	1. Define gaps in knowledge within regions, led primarily by the communities living there by introducing a consultation period of communication with neglected indigenous communities.
	2. Support #1 with pilot information via construction of a literature review.
	3. Include social scientists in assessing perception of risk factors in local communities and conceptions of human-nonhuman animal interactions, using Knowledge, Attitudes, and Practices (KAP) as well as other techniques.

#### Opportunities for the development of biologicals in Latin America

Although different monoclonal antibodies have been clinically approved for bacterial and viral infections, monoclonal antibody-based therapies are not yet available for NTDs, including parasites and animal envenomations.^63^ High manufacturing costs and cross-reactivity of antibodies limit the development of these therapies. Therefore, a collaborative network of scientific research needs to be established, to develop and produce diagnostic kits, drugs and vaccine for neglected diseases. In this sense, institutes and centres of science and technology in the Americas have the necessary know-how and infrastructure to accompany this network, such as the Milstein Institute in Argentina, which has developed a molecular diagnostic test for Chagas disease for use in the neonatal population, approved for use in Argentina. The Latin American Network of Public Antivenom Manufacturing Laboratories (RELAPA, Red Latinoamericana de Laboratorios Públicos Productores de Antivenenos) aims to consolidate technical cooperation, research and training for the regional improvement of antivenom availability, under the coordination of the PAHO and its office Centro Panamericano de FiebreAftosa (Panaftosa).

#### Anthropological ideas

Since 2018, a renewed WHO-wide commitment to acting on determinants of health, and within that, broader social determinants and health equity, has emerged. The COVID-19 crisis has further drawn attention to social disparities, leading to changes in social demographics as people are forced to return back to rural communities. Therefore, an integrative approach for the future of these expanding communities living in the Amazon region, and who are susceptible to future pandemics, is needed. One of the innovative features of ATBRI is the inclusion of specialists in social sciences in field research, using quantitative and qualitative (including ethnographic) techniques, alongside human and veterinary medical specialists, biologists and ecologists, thereby creating new multidisciplinary research strategies.

### Usefulness of the ATBRI for individual participating agencies and the wider community

We propose to include non-governmental organisations, individual and private centres, the larger scientific community and, most importantly, those indigenous and riverside communities that are currently neglected and will benefit from the creation of this network. The identification of laboratories and their capacity to characterise, diagnose and process specific samples is necessary to build a collaborative approach to disease surveillance and control. Those participating in the ATBRI network will benefit from the involvement of research activities to expand diagnostic capabilities of Amazon regions for specific disease, as well as acquire training in veterinary, public and social sciences. The network will also support the identification of sustainable resource distribution and the associated limiting factors, provision and allocation of educational resources, pathogen repositories and the need for biologics for therapy.

## Conclusions

Historically, all the diseases and health conditions mentioned here have received little attention from public agencies and governments as well as the private sector. The acquisition of regional information on zoonotic NTDs will not only further progress on research but will also be imperative to inform healthcare decision-making and policy development for countries and communities across regions of the Amazon. The integration of different sectors through the ATBRI will not only advance the One Health approach but will provide a regional framework for effective collaboration.

## Data Availability

Not required.
